# Treatment by Tuberculin in General Practice

**Published:** 1909-03-20

**Authors:** J. W. Wandby Griffin


### THE GENERAL PRACTITIONER'S COLUMN.

[Contributions to this Column are invited, and if accepted will be paid for.]

### TREATMENT BY TUBERCULIN IN GENERAL PRACTICE.

By J. W. WANDBY GRIFFIN, M.A., M.B., B.C., M.R.C.S., L.R.C.P.

The curative value of tuberculin (Koch's T.R.) in tuberculous diseases has been recognised after fairly extended trial in hospitals; it is not so well known, however, that this agent may be of equal value in general practice, despite the different conditions there obtaining. The average medical practitioner has not the time nor energy necessary for the determination of the variations of the opsonic index, which was formerly considered an essential part of the scientific administration of this agent, in spite of the differences in results obtained in consequence of the personal equations of the investigators, and has to be content with clinical evidences of the progress of the disease; there can now be little doubt that these are sufficient in many cases to guide the administration of tuberculin with most satisfactory results.

The site of the disease is an important consideration in determining the advisability of this treatment. Tuberculin seems to be of most value in the chronic tuberculous lesions of bone where there are sinuses of long standing, and it has been recommended for tuberculous peritonitis and for diseases of the joints with careful graduated dosage. It seems to have but little efficacy for tuberculous glands in the neck, and to be very injurious in any but the smallest doses if there is, or has been, any tuberculous disease of the eyes; in this latter case an acute inflammation may be set up thus and grave consequences result. In acute or recently acute tuberculous lesions it seems to do more harm than good.

The following points should be borne in mind:—

1. The idiosyncrasy of the individual, though not marked as a rule, may, however, be so, and unless graduated dosage be employed excessive reaction occur. Children as a rule take tuberculin well.

2. The temperature is a valuable indication of the condition of the patient; a rise of two or three degrees within a day or two usually indicates that the dose has been excessive, although, however, not necessarily unbeneficial nor injurious; in some cases this evidence of reaction is delayed for a week or ten days.

3. The actual lesion also may exhibit evidences of reaction if the dose has been large; there is then redness and swelling round the opening of the sinus with some skin infiltration which persists usually for some days, after which the wound frequently looks more healthy, and healing continues more rapidly and effectively. Tuberculous joints occasionally react sharply to tuberculin, and considerable damage may be caused. Graduated dosage is, therefore essential in such until clinical evidences have established the optimum dose.

Tuberculin may be administered by hypodermic injection or by the mouth. An average hypodermic dose for sinuses of long standing is 1/2,000 mg. (old style); this is a very convenient dose to stock, since from it lesser quantities can be easily measured. Tuberculin must be quite freshly prepared unless the concentrated fluid retailed in 1 c.c. phials is used, which is said to keep well if stored in a cool and dark place; the process of dilution is, however, rather tedious. In many cases it is advisable to begin with a dose of 1/10,000 or 1/8,000 of a milligramme (old style) repeated at fortnightly intervals, the dose being gradually increased until a definite improvement appears—this dose being then taken as the standard for the individual. Rarely there may result some local infiltration of the tissues at the site of injection; this may persist a few days and be a little painful, but soon subsides without other adverse signs. Oral administration has some advantages over hypodermic; it is less alarming to the patient, and there is less danger of overdose and excessive reaction. Tablets are now made by Messrs. Allen and Hanbury at the suggestion of Dr. Heaton, of Westgate-on-Sea, which keep well and can be easily administered. Larger doses are necessary than in hypodermic treatment; 1/5,000 milligramme (old style) is a fair initial dose. The best time for taking tuberculin thus is probably one hour before breakfast; the tabloid should be crushed to powder, and the dose repeated on the two following mornings, and then discontinued for a fortnight. Given thus reaction occurs later than in hypodermic dosing, and is less marked, so that this method is safer for cases in which serous surfaces are involved. The opsonic index is affected by oral administration of tuberculin precisely as by hypodermic administration.

Tuberculin in cases of lupus is of marked value in conjunction with light treatment (ultra-violet rays): it shortens the necessary exposure and healing progresses more rapidly, smaller doses are advisable in such cases. Improvement has also been observed in lupus cases treated solely by tuberculin, although the benefit is slower and the dose required higher as a rule than in tuberculous bone diseases.

The data on which these opinions are formed were derived mainly from the patients at the Royal Sea-Bathing Hospital, Margate, under the care of Dr. Heaton, whom I have to thank for permission to publish these notes.

